# Apoptosis-Inducing Factor 2 (AIF-2) Mediates a Caspase-Independent Apoptotic Pathway in the Tropical Sea Cucumber (*Holothuria leucospilota*)

**DOI:** 10.3390/ijms23063008

**Published:** 2022-03-10

**Authors:** Xiaomin Li, Ting Chen, Xiaofen Wu, Xiao Jiang, Peng Luo, Zixuan E, Chaoqun Hu, Chunhua Ren

**Affiliations:** 1 CAS Key Laboratory of Tropical Marine Bio-Resources and Ecology (LMB), South China Sea Institute of Oceanology, Chinese Academy of Sciences, Guangzhou 510301, China; lixiaomin19@mails.ucas.ac.cn (X.L.); chan1010@scsio.ac.cn (T.C.); jiangxiao@scsio.ac.cn (X.J.); luopeng@scsio.ac.cn (P.L.); ezixuan20@mails.ucas.ac.cn (Z.E.); hucq@scsio.ac.cn (C.H.); 2 University of Chinese Academy of Sciences, Beijing 100049, China; 3 Southern Marine Science and Engineering Guangdong Laboratory (Guangzhou), Guangzhou 510301, China; 4 Institute for Integrative Biology of the Cell, University of Paris-Saclay, 91198 Paris, France; xiaofen.wu@i2bc.paris-saclay.fr

**Keywords:** apoptosis-inducing factor 2, cadmium, sea cucumber, apoptosis, caspase-independent

## Abstract

Apoptosis, also known as programmed cell death, is a biological process that is critical for embryonic development, organic differentiation, and tissue homeostasis of organisms. As an essential mitochondrial flavoprotein, the apoptosis-inducing factor (AIF) can directly mediate the caspase-independent mitochondrial apoptotic pathway. In this study, we identified and characterized a novel AIF-2 (*Hl*AIF-2) from the tropical sea cucumber *Holothuria leucospilota*. *Hl*AIF-2 contains a conserved Pyr_redox_2 domain and a putative C-terminal nuclear localization sequence (NLS) but lacks an N-terminal mitochondrial localization sequence (MLS). In addition, both NADH- and FAD-binding domains for oxidoreductase function are conserved in *Hl*AIF-2. *Hl*AIF-2 mRNA was ubiquitously detected in all tissues and increased significantly during larval development. The transcript expression of *Hl*AIF-2 was significantly upregulated after treatment with CdCl_2_, but not the pathogen-associated molecular patterns (PAMPs) in primary coelomocytes. In HEK293T cells, *Hl*AIF-2 protein was located in the cytoplasm and nucleus, and tended to transfer into the nucleus by CdCl_2_ incubation. Moreover, there was an overexpression of *Hl*AIF-2-induced apoptosis in HEK293T cells. As a whole, this study provides the first evidence for heavy metal-induced apoptosis mediated by AIF-2 in sea cucumbers, and it may contribute to increasing the basic knowledge of the caspase-independent apoptotic pathway in ancient echinoderm species.

## 1. Introduction

Apoptosis, also known as programmed cell death, is an essential biological process that plays critical roles in embryonic development, organic differentiation, and normal tissue homeostasis in metazoans [1]. During immune responses, apoptosis is also important in the cell-mediated killing mechanism for target cells that are invaded by pathogens [2]. The core functions of apoptosis are considered to be mediated by the classical intrinsic and extrinsic pathways with initiator and executioner caspases [3]. In addition, several caspase-independent apoptotic pathways play important roles in the immune system, including the release of apoptosis-inducing factor (AIF) from mitochondria, which can induce caspase-independent peripheral chromatin condensation and large-scale DNA fragmentation in the nucleus [4].

AIFs are essential mitochondrial flavoproteins with multiple cellular functions, including the maintenance of electron transport chain function, the regulation of reactive oxygen species (ROS) production, and the mediation of cell death [5]. AIFs are caspase-independent death effectors that may trigger chromatin condensation and DNA fragmentation to induce apoptosis [6]. The apoptotic functions of AIFs have been well confirmed in vertebrates [7]. The oxidoreductase activities of AIFs are performed by the small nicotinamide adenine dinucleotide (NADH)-binding domain within the larger flavin adenine dinucleotide (FAD)-binding domain [8,9]. However, regardless of the presence or absence of NADH and/or FAD, AIFs can induce nuclear apoptosis [8]. Among them, apoptosis-inducing factor 2 (AIF-2), also known as apoptosis-inducing factor-homologous mitochondrion-associated inducer of death (AMID) or ferroptosis suppressor protein 1 (FSP1), is ubiquitously found in either prokaryotes or eukaryotes [7,10,11]. AIF-2 belongs to the conserved pyridine nucleotide-disulphide oxidoreductase-2 (Pyr_redox_2) family in the Pfam database. In vertebrates, the mitochondrial localization sequence (MLS) that directs the protein to mitochondria is found in the amino-terminus of AIF-1 and AIF-3 but not AIF-2. Thus, AIF-2 is located in the outer mitochondrial membrane instead of in the mitochondrial intermembrane space, similar to AIF-1 and AIF-3 [4,12]. However, AIF-2 retains the C-terminal domain that contains a nuclear localization sequence (NLS) that directs the protein to the nucleus and a pro-apoptotic segment that can trigger apoptosis when it is activated [6]. Mitochondria play a key role in oxidative stress-induced apoptosis; among which, AIFs mainly exercise their functions by transferring from the mitochondrial membrane to the nucleus [13].

In mammalian cells, heavy metals, such as cadmium (Cd^2+^), cause oxidative stress in cells, and Cd^2+^-induced apoptosis is mediated by the activation of both caspase-dependent and AIF-mediated caspase-independent pathways [14,15]. In the kidney cells of grass carp, lipotoxic molecules, such as palmitic acid, cause endoplasmic reticulum stress by activating AIF-mediated apoptosis via the mitochondrial pathway [16]. In crustacean mud crabs and Pacific white shrimps, AIF participates in the immune response against white spot syndrome virus (WSSV) infection by inducing apoptosis of haemocytes [17,18]. However, knowledge is limited regarding caspase-independent apoptotic pathways in echinoderms, except in a case reported for the sea cucumber *Apostichopus japonicus*, in which AIF-1 could mediate apoptosis induced by heat stress with a negatively correlated expression of heat shock protein 70 (HSP70) [19].

The tropical sea cucumber (*Holothuria leucospilota*) is naturally distributed in the Indo-Pacific region, and can protect the seafloor environment by digesting the bottom organic debris and adjusting the seawater pH [20]. The artificial culture of *H. leucospilota* has been developed for future applications in the decontamination of marine environmental pollutants [21]. Mechanisms for the caspase-dependent extrinsic apoptotic pathway have been well investigated in *H. leucospilota*. In this case, the tumor necrosis factor receptor (TNF-R) that binds with its ligand has been shown to initiate this pathway [22], which may lead to the orderly activation of initiator caspase-8 and executioner caspase-6 [23,24] via Fas-associated death domain protein (FADD) [25]. This caspase-dependent apoptotic pathway has been found to be positively and negatively regulated by myeloid differentiation factor 88 (MyD88) and inhibitory kappa B kinase (IKK), respectively [26,27,28]. However, little is known about the caspase-independent apoptotic pathway in *H. leucospilota*, especially in response to environmental factors, such as Cd^2+^ stress. In the present study, the full-length cDNA of *H. leucospilota* AIF-2 (*Hl*AIF-2) was cloned, and its structure and phylogeny were characterized. Expression patterns of *Hl*AIF-2 were detected in various tissues and different embryonic and larval developmental stages, and in primary coelomocytes after challenge with the heavy metal Cd^2+^ and pathogen-associated molecular patterns (PAMPs). Furthermore, the involvement of *Hl*AIF-2 in apoptosis was investigated by its overexpression in HEK293T cells and its intracellular location and translocation in the absence and presence of Cd^2+^ stress.

## 2. Results

### 2.1. Molecular Cloning and Sequence Analysis of HlAIF-2

By using 3′-/5′-RACE approaches, the full-length cDNA sequence of *Hl*AIF-2 was obtained from *H. leucospilota* and deposited in GenBank under the accession number OM417064. The open reading frame (ORF) of *Hl*AIF-2 cDNA is 1119 bp in length and is predicted to encode a protein of 372 amino acids (a.a.) (Figure S1). The calculated molecular weight of *Hl*AIF-2 is 40.94 kDa, and the estimated isoelectric point is 5.50. *Hl*AIF-2 contains a putative C-terminal NLS (residues 291–322) but lacks an N-terminal MLS (Figure S1).

**Figure 1:**
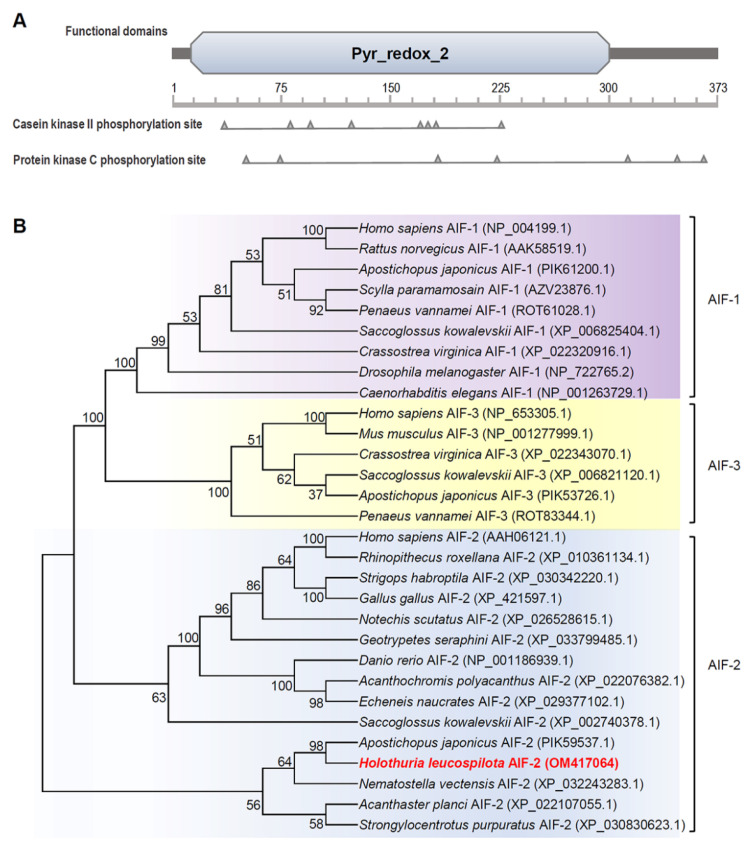
Functional domain and phylogenetic tree of *Hl*AIF-2. (**A**) Structural domain and active sites of *Hl*AIF-2 predicted using the SMART and ScanProsite programs. (**B**) Phylogenetic analysis of AIFs among various species using the neighbor-joining method with a bootstrap value of 1000.

Based on the SMART program, a conserved Pyr_redox_2 domain (residues 11–300) was predicted in the *Hl*AIF-2 a.a. sequence (Figure 1A). In addition, a casein kinase II phosphorylation site and a protein kinase C phosphorylation site were further indicated in the *Hl*AIF-2 a.a. sequence by the ScanProsite program (Figure 1A).

### 2.2. Phylogenetic, Homology and Structural Analysis

**Figure 2:**
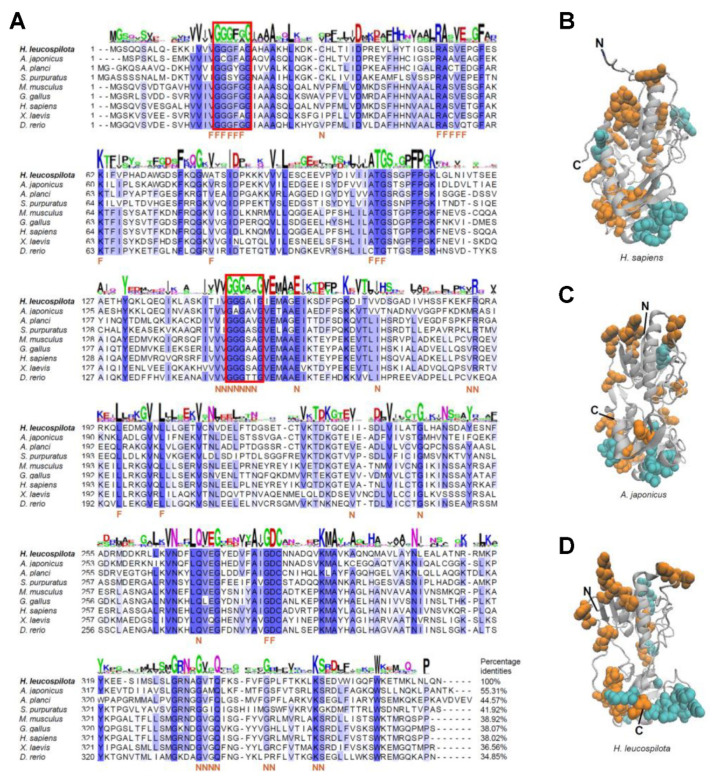
Sequence alignment and three-dimensional (3-D) structure of AIF-2 in different species. (**A**) A.a. sequence alignment of AIF-2 in nine Deuterostomia species. The conserved a.a. residues of *H. leucospilota* are presented in WebLogo format, and the conserved and similar a.a. residues between different species are labeled in dark blue and light blue, respectively. Residues that interact with FAD or NAD (in *H. leucospilota*) are marked as “F” or “N”, respectively. Two core consensus sequences of the typical motif “GXGXXG” are boxed in red lines. (**B**–**D**) Comparison of the 3-D protein of AIF-2 among human *H. sapiens* and the sea cucumber *A. japonicus* and *H. leucospilota*. Space-filling symbols indicate the most conserved binding sites of FAD (orange) and NAD (blue).

The results of the phylogenetic analysis revealed that AIFs from multiple animal species were classified into three branches: namely, AIF-1, AIF-2, and AIF-3 (Figure 1B). The branch of AIF-2s was further separated into two clades: vertebrate AIF-2s and invertebrate AIF-2s. Our newly identified *Hl*AIF-2 was found in the clade of invertebrate AIF-2s and shared a close evolutionary distance with the *A. japonicus* AIF-2 (Figure 1B). Multiple alignments of a.a. sequences showed that AIF-2s from different species in echinoderms and vertebrates shared considerably conserved sequences (Figure 2A). Most, if not all, a.a. which were supposed to interact with FAD and NADH were strongly conserved in AIFs, as precisely mapped in Figure 2A. Additionally, the core consensus for the typical motif GXGXXG was found at two distinct regions of the *Hl*AIF-2 a.a sequence (residues 17–22 and 148–153, Figure 2A). Three-dimensional (3-D) modeling was performed for the vertebrate AIF-2 from humans (*Homo sapiens*) and the echinoderm AIF-2s from the sea cucumber *A. japonicus* and *H. leucospilota*. As shown in Figure 2B–D, the probably conservative binding sites of NAD/FAD were highly comparable based on their 3-D structures.

### 2.3. Expression Patterns of HlAIF-2 among Different Tissues

**Figure 3:**
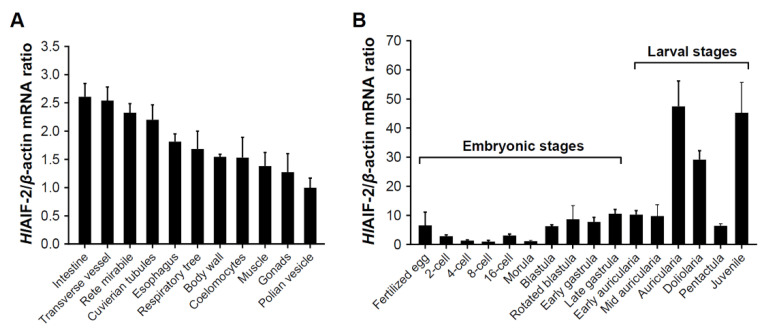
Expression patterns of *Hl*AIF-2. (**A**) Expression profile of *Hl*AIF-2 mRNA in different tissues. (**B**) Expression profiles of *Hl*AIF-2 mRNA during embryonic and larval stages. Data are presented as the mean ± SE (*n* = 3).

The mRNA expression pattern of *Hl*AIF-2 was analyzed in various tissues and different embryonic developmental stages by qPCR. As shown in Figure 3A, *Hl*AIF-2 mRNA was ubiquitously expressed in all the examined tissues, and the strongest expression was found in the intestine, followed by the transverse vessel, rete mirabile, Cuvierian tubules, esophagus, respiratory tree, body wall, coelomocytes, muscle, gonads, and polian vesicle (Figure 3A). However, the expression of *Hl*AIF-2 mRNA in the polian vesicle reached half of that in the transverse vessel (Figure 3A).

### 2.4. Expression Patterns of HlAIF-2 during Embryonic and Larval Development

As shown in Figure 3B, *Hl*AIF-2 mRNA was constitutively expressed in all detected embryonic and larval developmental stages, and the highest expression level was observed at the auricularia stage. After that, the expression level of *Hl*AIF-2 mRNA decreased sharply, reached its bottom at the pentactula stage, and increased again at the juvenile stage. Generally, *Hl*AIF-2 mRNA remained expressed at low levels in the embryonic stages but changed significantly in the larval stages.

### 2.5. HlAIF-2 Expression in Response to Challenges of CdCl_2_, LPS, and Poly (I:C)

**Figure 4:**
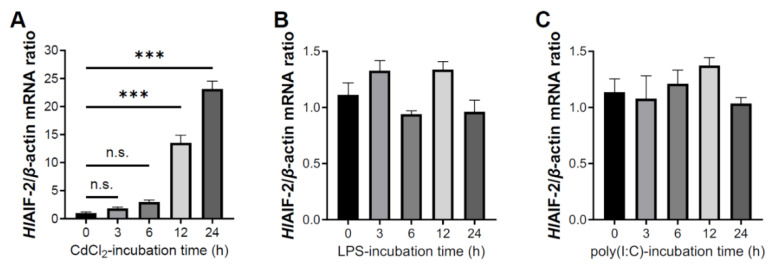
Expression analysis of HlAIF-2 after treatment of different exogenous stimulants. (**A**–**C**) Temporal expression pattern of *Hl*AIF-2 after treatment of CdCl_2_ (20 μM), LPS (10 μg/mL) smf poly (I:C) (10 μg/mL). Data are presented as mean ± SE (*n* = 3), and significant differences are analyzed using one-way ANOVA, and shown as n.s. *p* > 0.05 and *** *p* < 0.001.

Temporal expression of *Hl*AIF-2 mRNA in the coelomocytes was detected after challenge with cadmium chloride (CdCl_2_, 20 μM) as an oxidative stress (Figure 4A). After exposure to CdCl_2_, the expression of *HlAIF-2* was first upregulated with a 13.28-fold change (*p* < 0.001) at 12 h, followed by a 22.68-fold change (*p* < 0.001) at 24 h. In a parallel experiment, treatments with lipopolysaccharides (LPS) or polyriboinosinic polyribocytidylic acid [poly (I:C)] did not alter the expression level of *Hl*AIF-2 (Figure 4B,C).

### 2.6. Subcellular Localization of HlAIF-2 in HEK293T Cells

**Figure 5:**
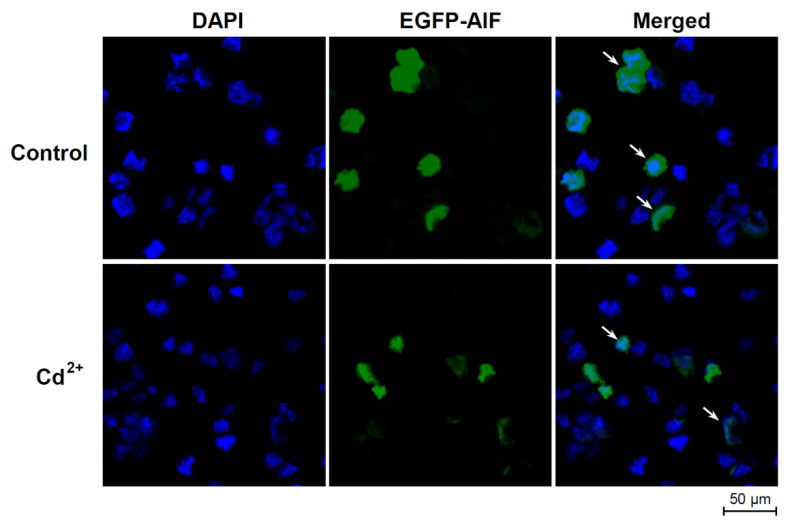
Subcellular localization of *Hl*AIF-2 in HEK293T cells. “DAPI” represents the DAPI-stained cell nuclei; “EGFP-AIF” represents the EGFP-labeled HlAIF-2 protein; “Merge” represents the combination of cell nuclei and *Hl*AIF-2 protein. Cd^2+^ treatment (12 h) could trigger *Hl*AIF-2 nuclear translocation, compared with the “Control” group. The arrows indicate the typical cells located in cytoplasm or translocated into nuclei.

The subcellular location of *Hl*AIF-2 was determined by transfection into HEK293T cells in the presence and absence of Cd^2+^. In the control group without Cd^2+^ treatment, *Hl*AIF-2 was located in both the cytoplasm and nucleus of the HEK293T cells. After incubation with CdCl_2_ for 24 h, part of the cytoplasmic *Hl*AIF-2 translocated into the nucleus (Figure 5), indicating that *Hl*AIF-2 tended to transfer into the nucleus during Cd^2+^-induced apoptosis.

### 2.7. Effects of HlAIF-2 Overexpression on Cell Apoptosis

**Figure 6:**
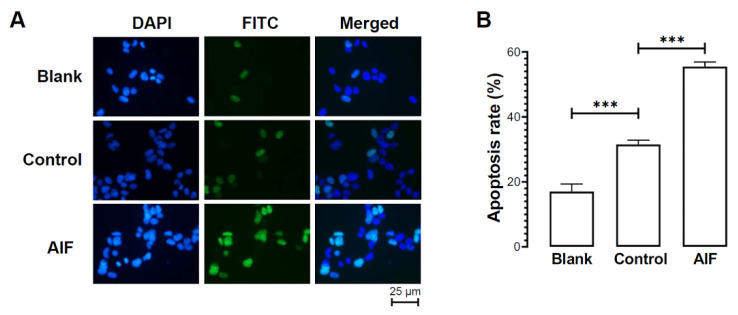
Apoptotic property of HEK293T cells transfected with pcDNA3.1/HA/*Hl*AIF-2 recombinant plasmid. (**A**) Detection of apoptosis by TUNEL assay. “DAPI” represents the DAPI-stained cell nuclei; “FITC” represents the FITC-stained fractured DNA fragments (marker for apoptosis); “Merged” represents the combination of cell nuclei and fractured DNA fragments. (**B**) Comparison of apoptosis rates for HEK293T cells in different groups. “Blank” represents the blank group (untransfected HEK293T cells); “Control” represents the control group (HEK293T cells transfected with pcDNA3.1/HA); “AIF” represents the experimental group (HEK293T cells transfected with pcDNA3.1/HA/*Hl*AIF-2). The values are expressed as mean ± SE (*n* = 3), and significant difference was analyzed by the Student’s *t*-test and shown as *** *p* < 0.001.

The function of *Hl*AIF-2 in the mediation of apoptosis was validated by transfection with the pcDNA3.1/HA/*Hl*AIF-2 plasmid in HEK293T cells. As shown in Figure 6, the apoptosis rate was detected by TUNEL assay. The results demonstrated that *Hl*AIF-2 overexpression could significantly induce apoptosis with DNA fragmentation in cell nuclei (Figure 6A), and the percentage of apoptosis was 17.05%, 31.54%, and 55.48% in the blank group, control group, and experimental group, respectively (Figure 6B).

## 3. Discussion

It is generally known that invertebrates lack adaptive immunity and so, as an alternative, innate immunity becomes a vital part of their immune system against invading pathogens and environmental stresses [29,30]. Apoptosis is a highly regulated and controlled process that confers advantages for organisms, and AIF is an ancient and conserved apoptotic executor that mediates apoptosis via a caspase-independent mitochondrial pathway [5,6].

Based on their widespread existence in various species, from invertebrates to humans [18,19,31,32], AIFs have been proposed to have an ancient and conserved pyridine nucleotide-disulfide oxidoreductase domain (Pyr_redox domain), which could generate superoxide rather than exhibit antioxidant activity [5]. Our current study found that *Hl*AIF-2 contained a Pyr_redox_2 domain and a deduced C-terminal NLS but lacked a recognizable MLS (Figure 1A). AIF precursors are usually synthesized in the cytoplasm and then imported into mitochondria through N-terminal MLS. However, in the presence of FAD, the MLS of AIF may be removed by proteolysis to produce mature AIF protein [4]. In contrast, AIF lacking MLS can spontaneously bind FAD and refold into mature AIF with a potential apoptosis-promoting function [4,8]. Normally, AIF performs mitochondrial functions and translocates to the nucleus only under the induction of apoptotic signals [33]. Once DNA damage occurs, the permeability of the mitochondrial outer membrane changes, and AIF is released from mitochondria [4,33]. However, subcellular localization experiments indicated that most *Hl*AIF-2 was localized in both the cytoplasm and nucleus of HEK293T cells without Cd^2+^ treatment (Figure 5). When Cd^2+^ was added, *Hl*AIF-2 in the cytoplasm tended to concentrate into the nucleus, indicating that overexpression of *Hl*AIF-2 may increase basic apoptosis. In addition, the similar phenomenon that Cd^2+^ treatment (12 h) could trigger AIF nuclear translocation dose-dependently has been observed in rat cells, previously [15,34].

Phylogenetic analysis showed that vertebrate AIF-2s first grouped with AIF-1s and AIF-3s, and then clustered into a branch of invertebrate AIF-2s (Figure 2B), illustrating that *Hl*AIF-2 is a fairly ancient gene with a conserved structure. The a.a. sequence of *Hl*AIF-2 shared high similarity with AIF-2s in other species (Figure 2A). AIFs are known for their oxidoreductase function, which is endowed by their NADH- and FAD-binding domains [35]. The Pyr_redox_2 domain is actually a smaller NADH-binding domain within a larger FAD-binding domain [9]. The binding sites of NADH and FAD in AIF-2s are highly conserved among different species (Figure 2A). Consistently, the 3-D structure of the *Hl*AIF-2 protein was highly comparable with those of AIF-2s from *H. sapiens* and *A. japonicus* (Figure 2B), including NADH- and FAD-binding domains.

Studies have shown that AIFs are widely distributed in various tissues in mammals [36,37]. In the present study, the transcripts of *HlAIF-2* were detected in all the tested tissues, with the highest expression level in the intestine (Figure 3A). Similarly, the intestine was the tissue with the highest expression level of *AIF* mRNA in Pacific white shrimps [18]. Previous studies showed that HSP70 could inhibit the nuclear translocation of AIFM1 during hibernation and thermal stimulation in *A. japonicus*, indicating a potential antiapoptotic response in the intestinal cells of sea cucumbers [19]. On the other hand, AIF-2 is reported to be involved in neural differentiation during embryonic development in vertebrates [37,38]. However, our present study showed that *HlAIF-2* expression continuously remained at a low level during embryonic development but increased significantly at the larval stages (Figure 3B). It is possible that the nerves of the sea cucumber initially formed at the larval stages, as was reported in *A. japonicus*, with the formation of five radial symmetrical nerve structures at the base of the oral tentacle [39,40]. In addition, the intestine of sea cucumbers gradually matures at the larval stages [41,42], and they need to accumulate nutrition for the transformation of planktonic to benthic lifestyles [21,43]. Hence, the roles of *Hl*AIF-2 in the embryonic and larval stages are speculated to be related to neurogenesis and intestinal development.

With the increasing global attention to marine’s sustainable development, an increasing number of studies are focused on the responses of marine animals to polluted marine environments. Among them, heavy metal (e.g., Cd^2+^) pollution will cause irreversible damage to plants, animals, aquatic life, and humans [30]. Cd^2+^ can induce mitochondrial oxidative stress and endoplasmic reticulum stress, consequently leading to apoptosis. Mitochondria release pro-apoptotic proteins via both the caspase-dependent pathways and the caspase-independent pathways, and the mechanism of Cd^2+^-induced apoptosis is believed to be complex [15]. In mammalian cells, oxidative stress caused by Cd^2+^ may induce the release of cytochrome c from mitochondria, followed by the activation of intracellular procaspase-9 protein, to form apoptosomes and produce active caspase-9 and caspase-3 proteins in a cascade, which ultimately induce apoptosis [44]. On the other hand, AIFs are released directly from the mitochondrial membrane and translocated to the nucleus to undergo apoptosis when mitochondria suffer from oxidative stress [45]. The mitochondrial apoptotic pathway has also been reported in the invertebrate Pacific oyster *Crassostrea gigas* [46]. In this study, we explored the caspase-independent mitochondrial apoptotic pathway that was induced by heavy metals in sea cucumbers. The expression of *Hl*AIF-2 in coelomocytes was significantly upregulated by challenge with Cd^2+^ (Figure 4A). Combined with subcellular localization experiments, showing that *Hl*AIF-2 concentrated from the cytoplasm to the nucleus during Cd^2+^ challenge, it is speculated that a conserved AIF-2-mediated apoptotic pathway could be induced by heavy metal stress in echinoderms.

To date, the roles of AIF-2 overexpression in the induction of apoptosis are still controversial. A previous study showed that overexpressed human AMID (AIF-2) could induce apoptosis in HEK293T cells in a dose-dependent manner, resulting in the condensation of chromatin and the formation of apoptotic bodies [10], while another study found that neither chromatin fragmentation nor protein translocation was observed with AMID overexpression [47]. Recent studies have shown that FSP1 (AIF-2) is an unrecognized anti-ferroptotic gene [11] that can inhibit the proliferation of lipid peroxides and prevent lipid damage and, consequently, ferroptosis [48]. The present study showed that overexpressed *Hl*AIF-2 could induce apoptosis after transfection into HEK293T cells (Figure 6), suggesting a conversed AIF-2-mediated apoptotic pathway in an ancient echinoderm model.

In conclusion, a novel AIF (*Hl*AIF-2) was identified from the sea cucumber *H. leucospilota* in this study. With its conserved functional domains and NLS, *Hl*AIF-2 can actively respond to Cd^2+^-induced oxidative stress in coelomocytes by translocation from the mitochondrial membrane into the nucleus. Moreover, the overexpressed *Hl*AIF-2 could induce apoptosis in HEK293T cells, with characteristics of DNA fragmentation in cell nuclei. These results collectively suggested that *Hl*AIF-2 was a conserved apoptotic executor that participated in the mitochondrial apoptotic pathway, that could be induced by heavy metal stresses. In addition, *Hl*AIF-2 mRNA was significantly upregulated during sea cucumber larval development and was expressed in the intestine with the highest expression level, suggesting its role in neurogenesis and intestinal development. Given that apoptosis is a complicated mechanism that can be mediated by multiple pathways, the conservation and specificities of apoptosis in echinoderms still need to be further investigated.

## 4. Materials and Methods

### 4.1. Animals and Tissue Collection

Healthy tropical sea cucumbers (*H. leucospilota*) weighing 100 ± 10 g were obtained from Daya Bay (Shenzhen, China) and temporarily reared in an aquarium with filtrated and aerated seawater (salinity of 35‰ and temperature of 30 °C) for a week before experiments. Sea cucumbers were dissected on ice, and the tissues were collected, frozen rapidly in liquid nitrogen, and stored at −80 °C until RNA extraction. The coelomic fluids were centrifuged at 1000× *g* for 10 min at 4 °C to harvest the coelomocytes, which were then kept in 1 mL of TRIzol reagent (Invitrogen, Carlsbad, CA, USA) at −80 °C. All animal experiments were conducted in accordance with the guidelines of the South China Sea Institute of Oceanology, Chinese Academy of Sciences, and this research does not contain any studies with human participants.

### 4.2. Molecular Cloning of HlAIF-2 Full-Length cDNA

Total RNA from the intestine of *H. leucospilota* was extracted using TRIzol reagent (Invitrogen) and reverse-transcribed to synthesize first-strand cDNA, using the PrimeScript™ II 1st Strand cDNA Synthesis Kit (Takara, Kusatsu, Japan). To obtain corresponding full-length cDNA sequences, 3′- and 5′-rapid amplification of cDNA ends (RACE) was performed using the 3′ Full Race Core Set Ver. 2.0 and 5′ Full Race Kit (Takara) with gene-specific primers (3′ RACE1/3′ RACE2 and 5′ RACE1/5′ RACE2, Table S1), respectively, which were designed based on a partial sequence for the *Hl*AIF-2 homolog from a transcriptomic library of *H. leucospilota* coelomocytes previously constructed by our lab [49].

### 4.3. Bioinformatics Analysis

Open reading frame (ORF) and a.a. sequences were deduced using ORF Finder (https://www.ncbi.nlm.nih.gov/orffinder/, accessed on 20 August 2021). Structural domains were predicted using the SMART (http://smart.embl-heidelberg.de/, accessed on 30 September 2021) and ScanProsite (http://prosite.expasy.org/, accessed on 30 September 2021) programs. A phylogenetic tree was constructed based on the a.a. difference (p-distance) with the neighbor-joining method (pairwise deletion) with 1000 bootstrap replicates using MEGA X (downloaded from http://www.megasoftware.net/, accessed on 7 October 2021. Alignment for a.a. sequences among various species was performed with the Clustal Omega program (http://www.ebi.ac.uk/Tools/msa/clustalo/, accessed on 7 October 2021) and demonstrated using the Jalview program (http://www.jalview.org/, accessed on 7 October 2021). Three-dimensional (3-D) models were deduced with Swiss modeling software provided by the SWISS-MODEL server (http://swissmodel.expasy.org/, accessed on 10 January 2022) and visualized by the VDM program (http://www.ks.uiuc.edu/Research/vmd/, accessed on 10 January 2022).

### 4.4. Tissue Distribution and Ontogeny of HlAIF-2 mRNA Expression

The tissue distribution of *Hl*AIF-2 mRNA was quantitatively detected in three individuals, and the selected tissues included coelomocytes, intestine, outer body wall, respiratory tree, rete mirabile, transverse vessel, polian vesicles, muscle (longitudinal muscle bands), esophagus, Cuvierian tubules, and gonads, as previously described [24]. Embryos and larvae of *H. leucospilota* were sampled in different developmental stages according to their morphologies [42], including fertilized egg, 2-cell, 4-cell, 8-cell, 16-cell, morula, blastula, rotated blastula, early-gastrula, late-gastrula, early-auricularia, mid-auricularia, auricularia, doliolaria, pentactula, and juvenile.

### 4.5. Primary Culture and Challenge of Coelomocytes

Sea cucumber primary coelomocytes were prepared as previously described [49]. After culture at 28 °C for 18 h in Leibovitz’s L-15 culture medium (Invitrogen), coelomocytes were challenged with CdCl_2_ (20 μM), LPS (10 μg/mL) or poly (I:C) (10 μg/mL), and the cells were harvested at 0, 3, 6, 12, and 24 h after administration.

### 4.6. Detection of HlAIF-2 Transcript by Real-Time PCR

Total RNA was extracted with TRIzol reagent, digested with gDNA Eraser (Takara), and reverse-transcribed using the PrimeScript™ RT Reagent Kit (Takara) for quantitative PCR (qPCR). Specific primers Q*Hl*AIF-2-F and Q*Hl*AIF-2-R (Table S1) were designed based on the obtained *Hl*AIF-2 cDNA sequences. qPCRs were performed using SYBR Premix Ex Taq™ II (Takara) in a final volume of 20 μL, with the conditions of 40 cycles of 95 °C for 5 s and 60 °C for 30 s. In this experiment, *Hlβ*-actin was used as an internal control to verify qPCR results.

### 4.7. Plasmid Construction, Cell Line Culture and Transfection

The coding region of *Hl*AIF-2 was amplified by PCR, using the gene-specific primers P*Hl*AIF-2-F and P*Hl*AIF-2-R (Table S1) and subcloned into the expression vectors pEGFP-N1 (Promega, Madison, WI, USA) and pcDNA3.1/HA (Invitrogen) by homologous recombination using a Hieff Clone Plus One Step Cloning Kit (Yeasen, Shanghai, China). All the plasmids used for transfection were extracted from overnight bacterial cultures using a Plasmid MiniPrep DNA Kit (Axygen, Union City, CA, USA), and all constructed recombinant plasmids were subsequently verified by DNA sequencing.

HEK293T cells were seeded in a 6-well plate and cultured in Dulbecco’s modified Eagle’s medium (HyClone, Logan, UT, USA) containing 10 % fetal calf serum (FCS), penicillin (100 μg/mL), and streptomycin (100 μg/mL) at 37 °C with 5 % CO_2_ for 24 h. Then, pEGFP-N1/*Hl*AIF-2 plasmid (2 μg/well) and pcDNA3.1/HA/*Hl*AIF-2 plasmid (2 μg/well) were transfected into HEK293 cells using 3 μL of Lipofectamine 2000 (Invitrogen). In parallel, the pcDNA3.1/HA blank plasmid was transfected into HEK293 cells as a control.

### 4.8. Subcellular Localization and Translocation

After transfection for 24 h, HEK293 cells transfected with pEGFP-N1/*Hl*AIF-2 plasmid were, then, treated with CdCl_2_ (20 μM) for 12 h. As a control, HEK293 cells were transfected with pEGFP-N1/*Hl*AIF-2 plasmid and cultured for 36 h without CdCl_2_ treatment. Subsequently, HEK293 cells were rinsed with PBS for 5 min, fixed with precooled 4% paraformaldehyde for 10 min, rinsed again with PBS for 5 min, treated with 0.5% Triton X-100 for 10 min, and stained with DAPI (1 mg/mL) for 10 min in the dark. Finally, HEK293 cells transfected with fluorescent vectors were directly observed by a confocal fluorescence microscope (Leica, Wetzlar, Germany).

### 4.9. Detection of Apoptosis

After transfection for 4 h, the untransfected HEK293 cells (blank group) and HEK293 cells that were transfected with pcDNA3.1/HA (control group) or pcDNA3.1/HA/*Hl*AIF-2 (experimental group) were cultured in 10 mL of DMEM containing 10 % FCS at 37 °C with 5 % CO_2_ for 48 h. Then, the apoptotic cells in the three groups were observed by a terminal deoxynucleotidyl transferase (TdT)-mediated dUTP nick-end labeling (TUNEL) assay, as described previously [23].

### 4.10. Data Transformation and Statistical Analysis

All data are presented as the mean ± standard error (SEM). Statistical analysis was performed using one-way ANOVA followed by Fisher’s least significant difference (LSD) test with SPSS 22.0 (IBM Software, Armonk, NY, USA), and statistical significance was determined at n.s. *p* > 0.05, * *p* < 0.05, ** *p* < 0.01, and *** *p* < 0.001.

## Data Availability

The data presented in this study are available in the article.

## Supplementary Materials

The following supporting information can be downloaded at: https://www.mdpi.com/article/10.3390/ijms23063008/s1.
