# Supplementary material for: Apoptosis-Inducing Factor 2 (AIF-2) Mediates a Caspase-Independent Apoptotic Pathway in the Tropical Sea Cucumber (Holothuria leucospilota)
Source: Int J Mol Sci. 2022 Mar 10;23(6):3008. doi: 10.3390/ijms23063008 (PMC8954137; doi:10.3390/ijms23063008)
Supplement: Supplementary file 1 [file ijms-23-03008-s001.zip › ijms-1595358-supplementary.pdf]

International Journal of *Molecular Sciences*

MDPI

Article

Apoptosis-Inducing Factor 2 (AIF-2) Mediates a Caspase-Independent Apoptotic Pathway in the Tropical Sea Cucumber (*Holothuria leucospilota*)

Xiaomin Li [1,2], Ting Chen [1,3], Xiaofen Wu [4], Xiao Jiang [1,3], Peng Luo [1,3], Zixuan E [1,2], Chaoqun Hu [1,3] and Chunhua Ren [1,3,*]

[1] CAS Key Laboratory of Tropical Marine Bio-Resources and Ecology (LMB), South China Sea Institute of Oceanology, Chinese Academy of Sciences, Guangzhou 510301, China; lixiaomin19@mails.ucas.ac.cn (X.L.); chan1010@scsio.ac.cn (T.C.); jiangxiao@scsio.ac.cn (X.J.); luopeng@scsio.ac.cn (P.L.); ezixuan20@mails.ucas.ac.cn (Z.E.); hucq@scsio.ac.cn (C.H.)

[2] University of Chinese Academy of Sciences, Beijing 100049, China

[3] Southern Marine Science and Engineering Guangdong Laboratory (Guangzhou), Guangzhou 510301, China

[4] Institute for Integrative Biology of the Cell, University of Paris-Saclay, Paris 91198, France; xiaofen.wu@i2bc.paris-saclay.fr

* Correspondence: rosemary166@sina.com

Supplementary Materials

Table S1. Primer sequences used in this study.

Name	Sequence (5' - 3')
**For sequence verification**	
*Hl*AIF-2-F	GTCATCTCACAATCATAGACCCTC
*Hl*AIF-2-R	GACCGATCCAAACATCCTCA
**For cDNA cloning**	
3' RACE1	TCGGAACCATCTGTAAACAACT
3' RACE2	TCACCGATGGCAAAGACGTCTT
3' RACE3	TGGACTCTTCCTTGTAAGGCTT
5' RACE1	TTGTCCTAGAGAGTTGCGAGGA
5' RACE2	GGAGTACCTGCACTATACCATT
**For recombinant plasmid construction**	
P*Hl*AIF-2-F	CGC<u>GGATCC</u>ATGGGAAGCCAACAGTCAG CTCTGC
P*Hl*AIF-2-R	GGTG<u>CTCGAC</u>TCAGTTCTGCAGATTAAGC TTCA
**For qPCR**	
Q*Hl*AIF-2-F	CGACAAAGGGCTCGGAAAC
Q*Hl*AIF-2-R	CTCGGAACCATCTGTAAACAACTC
Hlβ-actin-F	CCAGAGGAACACCCAGTC
Hlβ-actin-R	AGGGCGTAACCTTCATAG

```
   1                  cacagacagaccacagaaaatgagaaaacctacctgcctctagcgtctaacgttatttttagttctgtgg
  74 agattcggggaccgctgaatcaggaccaatgtctgctggagttctgtccgtcaacagtctatagtaaattatacagggttgtttacc
 164 atgggaagccaacagtcagctctgcaggagaagaagattgtagttgtaggaggtgggtttgctggagcacatgctgccaagcatttg
   1  M  G  S  Q  Q  S  A  L  Q  E  K  K  I  V  V  V  G  G  G  F  A  G  A  H  A  A  K  H  L
 254 gacaaatgtcatctcacaatcatagaccctcgggagtacctgcactataccattggtagtctcagggcatctgtagagccaggtttt
  31  D  K  C  H  L  T  I  I  D  P  R  E  Y  L  H  Y  T  I  G  S  L  R  A  S  V  E  P  G  F
 344 agcaagattttgtgccacatgctgatgcttggggagacagtttcaagcaaggatgggccacatcaatagatcccaagaagaaggtt
  61  S  K  I  F  V  P  H  A  D  A  W  G  D  S  F  K  Q  G  W  A  T  S  I  D  P  K  K  K  V
 434 gtcctagagagttgcgaggaggtgccatatgacattgtaatcatcgcgacggggagcagcggaccatttccaggaaaactgggtttg
  91  V  L  E  S  C  E  E  V  P  Y  D  I  V  I  I  A  T  G  S  S  G  P  F  P  G  K  L  G  L
 524 atagtcacatccgaggaagcagaaactcattaccaaaagctgcaagaacagatcaaattggcatcaaagataactatagttggtggt
 121  I  V  T  S  E  E  A  E  T  H  Y  Q  K  L  Q  E  Q  I  K  L  A  S  K  I  T  I  V  G  G
 614 gcaattggcattgaaatggctggagaaatcaaatcagacttcccaggaaaggatatcacagttgtggacagtggtgctgatatcgtc
 151  A  I  G  I  E  M  A  G  E  I  K  S  D  F  P  G  K  D  I  T  V  V  D  S  G  A  D  I  V
 704 agttcctttaaagagaaatttcgacaaagggctcggaaacagctggaggatatgggtgtgaatctgttattaggggagactgtttgt
 181  S  S  F  K  E  K  F  R  Q  R  A  R  K  Q  L  E  D  M  G  V  N  L  L  L  G  E  T  V  C
 794 gtggatgagttgtttacagatggttccgagacttgtacagtgaagacagatactggacaggagattatatccgatctagttatcctt
 211  V  D  E  L  F  T  D  G  S  E  T  C  T  V  K  T  D  T  G  Q  E  I  I  S  D  L  V  I  L
 884 actggcttacatgccaattcagatgcctatgaaagtaatttgctgacagaatggatgacaaacgattattgaaagtgaacgatttc
 241  T  G  L  H  A  N  S  D  A  Y  E  S  N  F  A  D  R  M  D  D  K  R  L  L  K  V  N  D  F
 974 caggtggaagggtatgaagacgtctttgccatcggtgactgcaataatgccgatcaagtgaaaatggctgtcaaggcacaaaatcaa
 271  Q  V  E  G  Y  E  D  V  F  A  I  G  D  C  N  N  A  D  Q  V  K  M  A  V  K  A  Q  N  Q
1064 gcagtattggcttataacttggaagcactagccaccaacaggagaatgaagccttacaaggaagagtccattatgagcttgtctctt
 301  A  V  L  A  Y  N  L  E  A  L  A  T  N  R  R  M  K  P  Y  K  E  E  S  I  M  S  L  S  L
1154 agaaatgcgggagtgacacagtttaagtcttttgtctttggtcctctgttcacaaagaaactgaagagtgaggatgtttggatcggt
 331  R  N  A  G  V  T  Q  F  K  S  F  V  F  G  P  L  F  T  K  K  L  K  S  E  D  V  W  I  G
1244 ttctggaaagaaacgatgaagcttaatctgcagaactgagaattgcaagttctcaaaacaacaatttgaaaggatgcagactgcagg
 361  F  W  K  E  T  M  K  L  N  L  Q  N  *
1334 tgagcaagtaaatggaagtagtgaagaatagaaattagagagtcagcagttcaatcccatcccgttcaaagaaaggtgcatttgtat
1424 cttttcttttaaaaaaaaaaaaaaaaaaaaaaaaaaaaaaaa
```

.

Figure S1. Nucleotide and deduced a.a. sequences of *Hl*AIF-2. The Pyr_redox_2 domain is marked in gray shade, and the nuclear localization sequence (NLS) is boxed in red lines.
